# Bioactive Hybrids Containing Artificial Cell Membranes and Phyto-Gold–Silver Chloride Bio-Nanoparticles

**DOI:** 10.3390/ijms252211929

**Published:** 2024-11-06

**Authors:** Marcela-Elisabeta Barbinta-Patrascu, Cornelia Nichita, Monica Enculescu, Valentin-Adrian Maraloiu, Mihaela Bacalum, Camelia Ungureanu, Catalin Constantin Negrila, Irina Zgura

**Affiliations:** 1 Department of Electricity, Solid-State Physics and Biophysics, Faculty of Physics, University of Bucharest, 405 Atomistilor Street, P.O. Box MG-11, 077125 Magurele, Romania; marcela.barbinta@unibuc.ro; 2 CTT-3Nano-SAE Research Center, Faculty of Physics, University of Bucharest, ICUB, 405 Atomistilor Street, P.O. Box MG-38, 077125 Magurele, Romania; 3 National Institute for Chemical-Pharmaceutical Research and Development, 112 Vitan Avenue, 031299 Bucharest, Romania; 4 National Institute of Materials Physics, Atomistilor 405A, 077125 Magurele, Romania; mdatcu@infim.ro (M.E.); maraloiu@infim.ro (V.-A.M.); catalin.negrila@infim.ro (C.C.N.); 5 Department of Life and Environmental Physics, Horia Hulubei National Institute for Physics and Nuclear Engineering, Reactorului, 30, 077125 Magurele, Romania; bmihaela@nipne.ro; 6 Department of General Chemistry, Faculty of Chemical Engineering and Biotechnologies, The National University of Science and Technology POLITEHNICA Bucharest, Gheorghe Polizu 1-7 Street, 011061 Bucharest, Romania; ungureanucamelia@gmail.com

**Keywords:** gold–silver chloride nanoparticles, phytosynthesis, *Achillea millefolium* L.; antioxidant activity, antimicrobial properties, antiproliferative activity

## Abstract

This research targets the need for eco-friendly strategies in the synthesis of bioactive materials, addressing the importance of valorization of vegetal waste. This study focuses on developing biohybrids containing biomimetic lipid vesicles and phytosynthesized gold–silver chloride nanoparticles (AuAgCl NPs) derived from *Achillea millefolium* L. extract. By leveraging the natural antioxidant and antimicrobial properties of the plant, the research proposes a sustainable approach to creating materials with potential biomedical applications. The biomimetic membranes were loaded with chlorophyll *a*, a natural spectral marker. Three types of bioactive materials (biohybrids) were developed by varying the lipid vesicle/AuAgCl NP ratio. Optical (UV-Vis, fluorescence emission, FTIR), structural (XRD), elemental (EDX, XPS), and morphological (TEM) studies were performed to characterize the bio-developed materials. The hydrophobic/hydrophilic characteristics of the samples were investigated by measuring the water contact angle, and their size was estimated by DLS and TEM. Zeta potential measurements were used to evaluate the physical stability of phyto-developed particles. Antioxidant properties of phyto-particles were investigated through the chemiluminescence technique. The obtained biomaterials exhibited high antioxidant activity and antiproliferative activity against HT-29 and B-16 cancer cells. Therapeutic index values were calculated for each biohybrid. Additionally, the bio-prepared hybrids revealed biocidal action against *Staphylococcus aureus* and *Enterococcus faecalis*. The phyto-developed biomaterials are promising in biomedical applications, particularly as adjuvants in cancer therapy.

## 1. Introduction

Rapid progress in all research areas and technologies has an impact on the environment, especially concerning the synthesis of (nano)materials. Therefore, ecological strategies need to be addressed for the development of new materials with wide applicability.

This research work presents a “green” strategy to develop biohybrids containing bimetallic phyto-nanostructures (AuAgCl NPs) and biomimetic cell membranes labeled with a natural spectral marker, chlorophyll, with promising potential in bio-applications. AuAgCl NPs have been obtained from an aqueous extract of aerial parts (inflorescences) of *Achillea millefolium* L. (commonly named yarrow). *A. millefolium* is an important medicinal plant belonging to the Asteraceae family with a long history of use, beginning in ancient times when it was used in the form of herbal tea or as a wound-healing remedy [1]. In the Middle Ages, this plant was known as “soldier’s grass” due to its use in wound treating [2]. Yarrow has been widely used in the treatment of various diseases, including gastrointestinal diseases and diuretic and analgesic applications, as well as for the treatment of bruises, pulmonary disorders, inflammation, respiratory ailments (such as asthma and bronchitis), headaches, dyspepsia, skin inflammation, urinary and hepato-biliary disorders, and overactive cardiovascular conditions [2,3,4,5]. Its bioactivities/therapeutical effects (antioxidant, antimicrobial, anti-inflammatory, and antiproliferative properties) are due to its chemical composition, which includes active compounds such as flavonoids, phenolic acids (caffeic, cinnamic, and benzoic acid derivatives), terpenes (guaianolides, diterpenes, sesquiterpenes), phytosterols, organic acids, fatty acids, and alcohols [2,6,7].

AuAgCl NPs were synthesized using a Green Chemistry approach, leveraging the natural reducing agents found in the yarrow extract. Gold–silver chloride nanoparticles exhibit unique optical properties, significant surface reactivity, and enhanced biological compatibility. The combination of gold and silver at the nanoscale can result in synergistic effects that improve their antimicrobial and anticancer activities. The chlorophyll present in the extract not only facilitates the synthesis but also enhances the stability and functionality of the nanoparticles [8,9]. Biomimetic cell membranes are synthetic constructs designed to mimic the natural properties of biological membranes. These artificial membranes are essential in studying cell interactions, drug delivery systems, and biosensor development. By incorporating chlorophyll as a natural spectral marker, these membranes can be used to track and analyze interactions with nanoparticles and biological systems [10]. Chlorophyll provides an environmentally friendly and biocompatible labeling method, enhancing the overall sustainability of hybrid materials [11,12,13,14,15].

This research is original and brings to the forefront the idea of harnessing natural resources—plants—to create new materials with unexpected properties. No previous studies have reported the development and biophysical characterization of “green” biohybrids containing yarrow-derived AuAgCl NPs and chlorophyll-labeled artificial cell membranes. This novel approach paves the way for future research in the field of nanotechnology and biomedicine, potentially leading to innovative treatments and diagnostic tools. The biophysical characterization of these biohybrids, including their structural, optical, and functional properties, will provide valuable insights into their potential uses in various medical and technological fields. By exploring the interface between natural products and advanced materials, this study highlights the importance of interdisciplinary approaches in achieving sustainable and impactful scientific advancements.

The potential applications of these biohybrids are vast and promising, spanning various fields including biomedicine, environmental science, and nanotechnology. In biomedicine, the unique properties of metallic nanoparticles can be harnessed for targeted drug delivery, enhancing the efficacy and specificity of treatments while minimizing side effects. Additionally, their antimicrobial and anticancer activities make them suitable for developing new therapeutic agents and diagnostic tools [16,17].

The chlorophyll-labeled artificial cell membranes can be utilized in biosensors to detect and monitor biological and chemical changes in real time, providing critical data for medical diagnostics and environmental monitoring. These biosensors could be particularly valuable in detecting pathogens or pollutants, contributing to public health and environmental protection [18].

The eco-friendly synthesis method and the use of natural materials align with the principles of Green Chemistry, promoting sustainability in material science. This approach could lead to the development of more sustainable manufacturing processes in the pharmaceutical and biotechnology industries.

The integration of natural and synthetic components in these biohybrids opens new avenues for research in biomimetics, potentially inspiring the creation of advanced materials with unprecedented functionalities.

## 2. Results and Discussion

### 2.1. Evaluation of Total Phenolic Content of Achillea-Derived Samples

Phenolic compounds are an interesting class of phyto-molecules bearing one or multiple hydroxyl groups on an aromatic ring, and they are responsible for scavenging free radicals, explaining their antioxidant properties and other bioactivities, such as antimicrobial properties, anti-inflammatory, and anticancer activities [19]. The TPC (total phenol content) values of the samples are presented in Table 1. The TPC value obtained in our study for the aqueous extract of *A. millefolium* flowers is close to the one reported by Eghdami et al., 48.4 ± 2.7 (mg/GAE·g^−1^) [20], and much higher than that reported by Mehmood et al., 16.34 ± 0.71 (mg/GAE·g^−1^) [21]. After the consecutive addition of appropriate amounts of AgNO_3_ and then HAuCl_4_ to the yarrow extract, the TPC value of the yarrow extract dropped drastically by 89.91%. This demonstrates that a considerable number of polyphenols was consumed in the phytosynthesis reaction of AuAgCl NPs, highlighting the important role of these phytocompounds in the development of nanoparticles using *Achillea millefolium* extract.

The TPC values of the biohybrids are lower than those of AuAgClNPs due to the dilution effect when the suspension of lipid vesicles that do not contain phenolic compounds was added.

### 2.2. Optical Characterization of Achillea-Derived Particles

**Figure 1:**
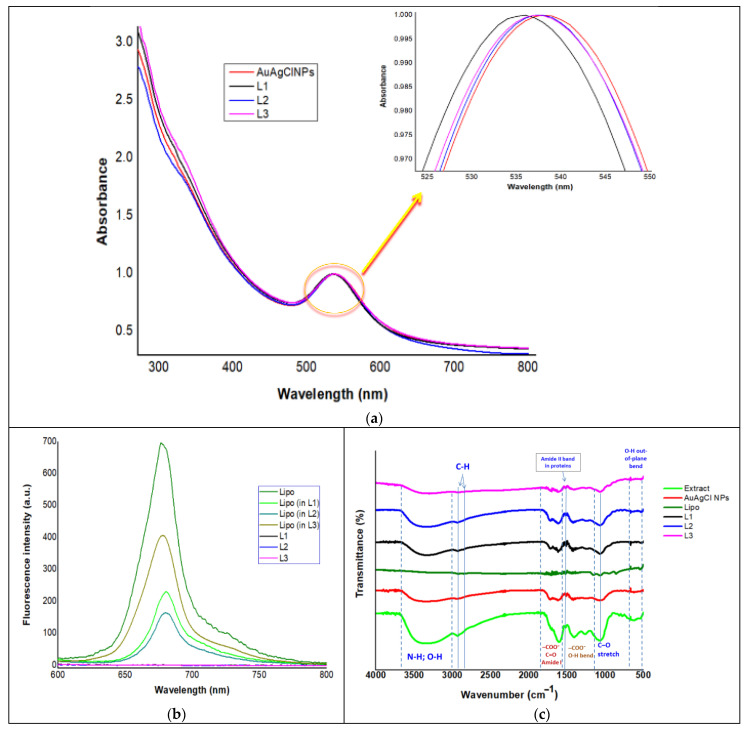
Optical characterization of *Achillea*-derived samples. (**a**) UV-Vis absorption spectra of yarrow-derived AuAgClNPs and the biohybrids (the spectra were normalized at their characteristic peak). (**b**) Fluorescence emission spectra of chlorophyll-containing samples (λ_excitation_ = 430 nm). The lipid vesicles are in the same concentrations as in the biohybrids. (**c**) Comparative presentation of FTIR spectra of the obtained samples.

The development of yarrow-derived NPs was first observed by means of UV–Vis absorption spectroscopy, as shown in Figure 1.

The plant-assisted synthesis of AuAgClNPs was confirmed by UV-Vis absorption spectroscopy. AuAgClNPs exhibited an intense peak at 538 nm, while the yarrow extract did not show any peak in the wavelength region of 500–600 nm (Figure S1, Supplementary Materials). The obtained biohybrids L1, L2, and L3 presented peaks at wavelengths of 536, 537.5, and 538.4 nm, respectively (Figure 1a). Our results are consistent with other scientific reports. For instance, Alti et al. reported the phytosynthesis of gold–silver bimetallic nanostructures from aqueous leaf extracts of coriander, soybean, and fenugreek, which showed peaks at wavelengths of 522, 533, and 541 nm, respectively [22].

Chlorophyll *a* embedded in artificial cell membranes was used as a natural fluorophor. The fluorescence emission spectra (Figure 1b) proved also the formation of biohybrids containing AuAgClNPs. The Chla-labelled biomimetic membranes showed an emission peak at 677 nm when excited at 430 nm. Fluorescently labelled biomimetic membranes undergo strong emission quenching after the addition of yarrow-generated AuAgCl NPs due to an energy transfer or an electron transfer process when the porphyrinic ring of Chla (loaded in the biomimetic membranes) directly binds to the metallic nanoparticle surface. These findings agree with other previous reports [23,24,25].

The FTIR results (Figure 1c) showed the presence on the NP surface of functional groups belonging to flavonoids, proteins responsible for the reduction in silver and gold ions and for the stability of developed nanoparticles. An intense sharp band centered at 3350, 3361, 3325, 3341, and 3341 cm^−1^ observed in the spectrum of the extract, AuAgClNPs, L1, L2, and L3, respectively, is assigned to the bending and stretching vibrations of hydroxyl groups intermolecularly hydrogen bonded in alcohols, phenolic compounds/polyphenols and polysaccharides, and the stretching vibrations of the primary and secondary amines [26]. The samples extract, AuAgClNPs, Lipo, L1, L2, and L3 showed FTIR bands attributed to a C–H anti-symmetric stretching vibration [27] located at wavenumbers 2930, 2925, 2922, 2924, 2931, and 2925 cm^−1^, respectively, and also the C–H symmetrical stretch vibration of alkyl chains [27] located at wavenumbers 2843, 2849, 2855, 2847, 2849, and 2847 cm^−1^, respectively. In addition, more bands appeared in the region of 700–500 cm^−1^ in the spectrum of extract, AuAgClNPs, and biohybrids (L1, L2, and L3), indicating the presence of hydroxyl groups [28] at the surface of the vegetal extract and of the yarrow-derived particles. The sharp band located at 857 cm^−1^ in Lipo, assigned to stretching vibrations of –CH_2_– [29], shifted to 877, 875, and 872 cm^−1^ in biohybrids L1, L2, and L3, respectively. This behavior agrees with our previous studies [24]. The sharp band at 945 cm^−1^, representing the antisymmetric stretching vibrations of the choline group (N^+^–(CH_3_)_3_) [24], which was identified in the Lipo spectrum, weakened in the FTIR spectra of biohybrids and shifted to 939 cm^−1^ in the L1 and L2 spectra and to 943 cm^−1^ in L3. The above-mentioned observations demonstrate the formation of biohybrids.

Other FTIR bands that are presented in the obtained yarrow-derived samples were due to the carbonyl stretch and carboxylate groups in proteins, the –C–O–C– ether, and the O–H bend (see Figure 1c).

The FTIR analysis indicates that the functional groups belonging to the phytomolecules of yarrow extract (such as proteins, polyphenols, alcohols, carboxylates, and ethers) acted as reducing and capping agents for AuAgClNPs. The presence of hydroxyls, carbonyls, carboxylates, ethers, and other functional groups on the surface of the yarrow-derived particles is also observed.

### 2.3. Evaluation of the Zeta Potential of the Phytometallic Particles

The zeta potential is an important parameter that evaluates the colloidal stability of a suspension. The ξ values are presented in Figure S2 (in the Supplementary Materials). All the samples displayed negative surface charge and moderate to good physical stability assured by repulsive forces between particles. The biomimetic lipid vesicles exhibited moderate stability (ξ_Lipo_ = −21.1 ± 7.82 mV), while the biohybrids showed greater stability compared to AuAgClNPs alone. The zeta potential values increased with the AuAgClNP content in the biohybrids. Thus, the most stable sample proved to be L2 (ξ_L2_ = −28.2 ± 4.44 mV). The zeta potential value of yarrow-derived AuAgClNPs (ξ_AuAgClNPs_ = –25.4 ± 4.37 mV) is in line with other zeta potential values for phytogenically metallic nanoparticles reported in the scientific literature. Thus, Hosny et al. prepared *T. capensis*—phytosynthesized AuNPs with a zeta potential value of −24.5 mV [30]. Mishra et al. reported the phytosynthesis of AgNPs and AuNPs from *Murraya koenigii* extract, with zeta potential values of −15.1 and −18.4 mV, respectively [31].

### 2.4. Structural Characterization

**Figure 2:**
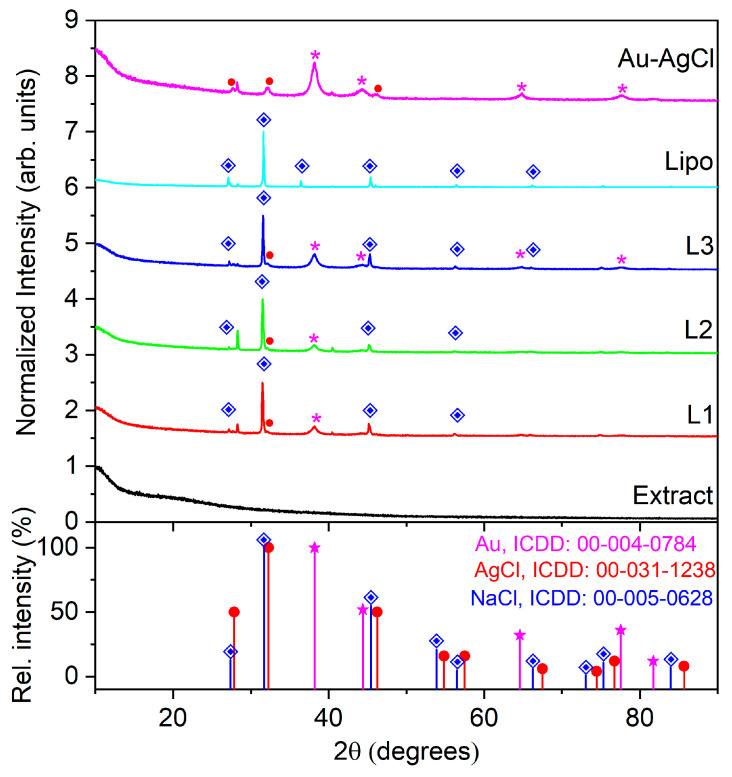
XRD patterns of the obtained samples. The diffraction peaks are marked using the symbols: ∗ (magenta) for Au; • (red) for AgCl; ◈ (blue) for NaCl.

The crystallinity of the samples was established by the diffraction peaks shown in Figure 2. It was found that the diffraction peaks positioned at (i) 38.2, 44.4, 64.6, and 77.5° correspond to the Miller indices of the reflecting planes (111), (200), (220), and (311) assigned to the gold in cubic phase (file 00-004-0784); (ii) 27.8, 32.2, and 46.2° correspond to the crystallographic planes (111), (200), and (220) specific to the cubic unit cell of the AgCl crystal (file 00-031-1238); and (iii) the diffraction peaks from 27.3, 31.7, 36.3, 45.4, 53.9, 56.5, and 66.2° correspond to the crystallographic planes (111), (200), (220), (311), (222), and (400) are attributed to the cubic phase of NaCl (file 00-005-0628). The XRD patterns corresponding to NaCl are presented in the samples containing biomimetic membranes due to the presence of a PBS buffer.

As observed, the yarrow extract is amorphous. The yarrow-derived AuAgCl NPs exhibit major peaks of Au and minor peaks of AgCl. The major peaks of Au are also visible in the XRD spectra of hybrids L1, L2, and L3. The diffraction peaks corresponding to the AgCl were not found in biohybrids L1, L2, and L3 because their preparation route involves the addition of an appropriate volume of liposomal suspension to a certain volume of plant-derived AuAgClNPs. As a result, the bio-derived bimetallic particle suspension is more diluted, and the minor peaks of AgCl are diminished. The formation of AgCl NPs was possible due to the presence of Cl^−^ in the soil and, consequently, in the plants, as Cl^−^ is an essential micronutrient for oxygenic photosynthetic organisms and is found in the environment at higher levels than those required by plants [32,33]. The presence of Cl^−^ in the yarrow extract was further demonstrated by the EDX spectrum (see Figure S5c, Supplementary Materials).

### 2.5. Estimation of Hydrodynamic Diameter

The average particle size (Zav) and the polydispersity index (PdI) of the samples were determined using dynamic light scattering measurements (Figure S3, Supplementary Materials). The lipid vesicles non-ultrasonicated presented an average size of 661 ± 48.54 nm (Figure S4, Supplementary Materials). DLS measurements revealed the nanoscaled size of the biogenic particles. The biohybrid systems ultrasonicated presented a mean hydrodynamic diameter of less than 200 nm, with PdI values ranging from 0.22 to 0.34, indicating relatively low dispersity. Our results regarding the size of phytometallic nanostructures are in line with other previous studies [34,35].

### 2.6. Morphological and Compositional Characterization

The features of the samples were investigated from a morphological and compositional point of view using the TEM, EDX, and XPS techniques.

TEM investigation revealed cell-like entities with multivesicular structures inside for the Chla- loaded liposomes (sample Lipo) (Figure S5g, Supplementary Materials).

For the extract sample, conventional TEM (CTEM) images (Figure S5a,b, Supplementary Materials) show areas with darker contrast with irregular shapes that were formed after suspension evaporated at room temperature. The content inside these shapes is amorphous. The EDX spectrum (Figure S5c, Supplementary Materials) shows the existence of several chemical elements: O, Na, Mg, Si, P, S, Cl, K, and Ca.

For the AuAgClNP sample, the CTEM image (Figure S5d, Supplementary Materials) reveals nanoparticles with several shapes/morphologies: circle, oval, triangle, and rod. The size varies from 7 nm to 110 nm. Similar shapes and sizes were obtained by Geethalakshmi and Sarada [36], who phytosynthesized metallic nanoparticles from *Trianthema decandra* root extract.

The (quasi)spherical shapes are predominantly because this shape is achieved by NPs to attain the minimum surface energy [35]. The interactions between the biomolecules arising from yarrow extract and stabilizing and capping the metallic NPs that developed are responsible for the different shape formations. Hosny et al. stated that the phytoconstituents present in the vegetal extract such as phenolics, flavonoids, alkaloids, terpenoids, glycosides, and tannins could have diverse reduction potentials that influence the bioreduction reaction, resulting in the development of metallic nanoparticles with multiple shapes [30].

In the high-resolution TEM (HRTEM) image (Figure S5e, Supplementary Materials), fringes can be observed, which demonstrate that nanoparticles are crystalline. The interplanar distances measured in the image help identify the crystalline structure. For cubic Au, fringes of 2.3 Å corresponding to the (111) plane, 2.0 Å to the (200) plane, and 1.4 Å to the (220) plane can be identified, while for cubic AgCl, only the fringe of 1.9 Å corresponding to the (220) plane is seen. The EDX spectrum (Figure S5f, Supplementary Materials) confirms the presence of Au, Ag, and Cl in the sample but also some other chemical elements: O, Na, Si, K, Ca, and Fe. The ratio between the Au peak and Ag peak suggests that Au nanoparticles are predominant.

As pointed out by Rai et al. [37] and by Geethalakshmi and Sarada [36], the presence of halide ions results in the development of phytogenically gold triangles using vegetal extract. In the present research work, NPs resembling triangles were obtained due to the presence of chloride ions in yarrow extract, as shown in Figure S5c (Supplementary Materials).

TEM investigation revealed cell-like entities with multivesicular structures inside (Figure S5g,h, Supplementary Materials) for the Chla-loaded liposomes (sample Lipo). The size of the liposomes varies between 715 nm and 2 µm. TEM images captured the fusion of lipid vesicles and the formation of larger ones. This happens frequently with lipid particles with simple composition, especially when they are deposited on solid supports. Inside the vesicle in Figure S5g (Supplementary Materials), small lipid vesicles between 80 and 120 nm are observed. In this sample, the chemical elements detected by EDX (Figure S5i, Supplementary Materials) are the same as the extract but in different concentrations.

**Figure 3:**
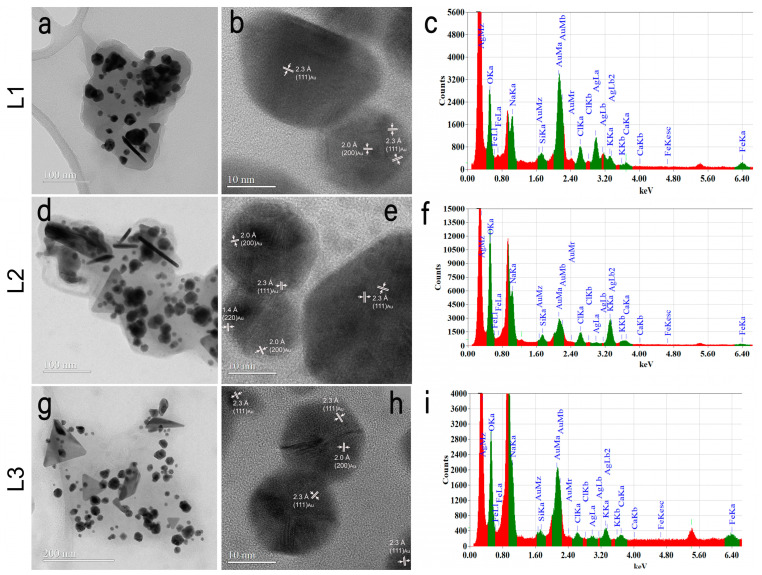
CTEM images (**a**,**d**,**g**), HRTEM images (**b**,**e**,**h**), and EDX spectra (**c**,**f**,**i**) obtained on the investigated samples.

In the case of samples L1, L2, and L3, CTEM images (Figure 3a,d,g) show that the morphologies and size of NPs do not change. They also demonstrate that biohybrids have the intended concentration of AuAgClNPs. In Figure 3g, corresponding to a concentration of AuAgClNPs of 33.3%, NPs are less agglomerated than in Figure 3d, corresponding to a concentration of AuAgClNPs of 66.7%. In HRTEM images (Figure 3b,e,h), the measured fringes correspond to the expected crystalline structures: cubic Au and cubic AgCl. Like in the case of AuAgClNPs, the EDX spectrum for L1, L2, and L3 samples (Figure 3c,f,i) contains the same chemical elements: Au, Ag, Cl, O, Na, Si, K, Ca, and Fe.

**Figure 4:**
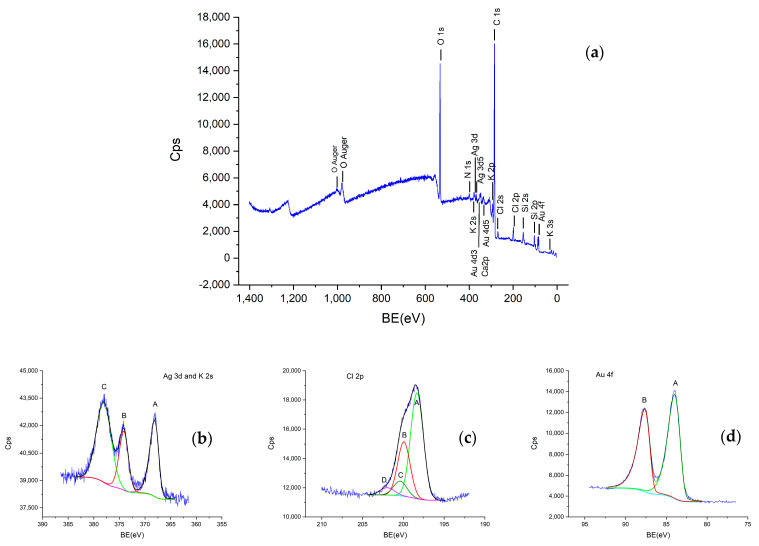
XPS spectra of the AuAgClNP sample: (**a**) survey spectrum; (**b**) Ag3s and K2s spectrum; (**c**) Cl2s spectrum; (**d**) Au4f spectrum.

The XPS results also confirm the formation of the AuAgCl nanoparticles. As shown in Figure 4a, the elements C, O, N, Si, K, Ca, Cl, Ag, and Au are clearly present, indicating the existence of Au and AgCl within the sample. In the high-resolution XPS spectrum presented in Figure 4b, three peaks located at 368.19 eV (peak A), 374.13 eV (peak B), and 378.04 eV (peak C) were found. Peaks A-B represent the Ag 3d doublet. The two lines have a doublet splitting of approximately 6 eV and an area ratio of about 3:2. The binding energy indicates AgCl, which is consistent with the literature [38,39]. Peak C represents K2s. In Figure 4c, the high-resolution XPS spectrum of Cl 2p is presented. Through fitting, the 2p^3/2^ (peak A, at 198.32 eV) and 2p^1/2^ (peak B, at 199.93 eV) doublets were identified, with a splitting of 1.6 eV and an area ratio of approximately 2:1. Peaks C-D indicate bonds with C in organic compounds. Peaks A-B indicate metal chlorides, including KCl and AgCl, which are difficult to differentiate, especially because the amount of Ag is very small compared to K. In Figure 4d, the high-resolution XPS spectrum of Au 4f is presented, consisting of two lines, Au 4f^7/2^ and Au 4f^5/2^, with a splitting of approximately 3.6–3.7 eV and an area ratio of about 4:3. The binding energy is very close to the ISO standard for metallic Au used for calibration (which is 83.96 eV). A bond with Cl would have produced peaks with binding energies above 84.4 eV. Therefore, the peaks at 83.99 eV and 87.65 eV correspond to metallic Au [38,40]. However, by correlating the results obtained from XPS with those from TEM and XRD, we can confirm the presence of silver chloride particles. This result is consistent with the literature [41].

### 2.7. The Wetting Properties of Achillea-Derived Samples

**Figure 5:**
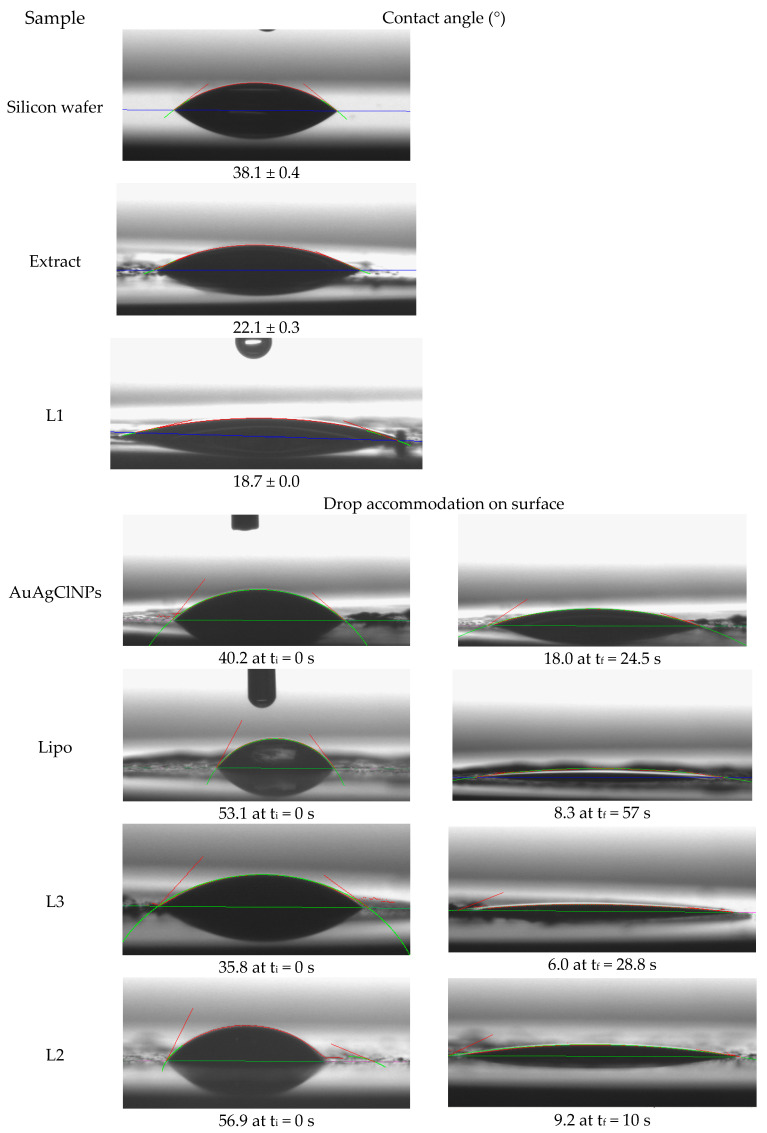
The wetting properties of *Achillea*-derived samples.

The wetting properties of *Achillea*-derived samples are presented in Figure 5.

In the case of the Lipo, L3, L2, and L1 samples, Cassie–Baxter’s type of wetting or even a Cassie impregnation was observed [42]. In the Cassie impregnation state, a liquid covers the surface texture such that a droplet sits above the solid–liquid composite surface [43,44].

An evolution of the water drop behavior was observed on the L2, L3, AuAgClNP, and Lipo samples (see Figure 5). Immediately after the water drop is deposited, it spreads over the surface, and the equilibrium value is reached within 10 to 60 s. The water drop settles on the surface, exhibiting Cassie-type wetting. In contrast, the contact angle values obtained for the L1 and extract samples are lower and do not show significant evolution, indicating a hydrophilic character. This hydrophilic nature obtained on all the samples suggests an increase in the adhesion in composites, enhanced biocompatibility in implant devices, and better liquid spreading properties [45]. Consequently, the improved bioactivities make these samples suitable for use in living systems as adjuvants in various therapies.

This investigation demonstrated the hydrophilic properties of the samples developed in this research, indicating their potential use as adjuvants in various therapies within living systems.

### 2.8. Evaluation of Biological Activities of Phyto-Particles

**Figure 6:**
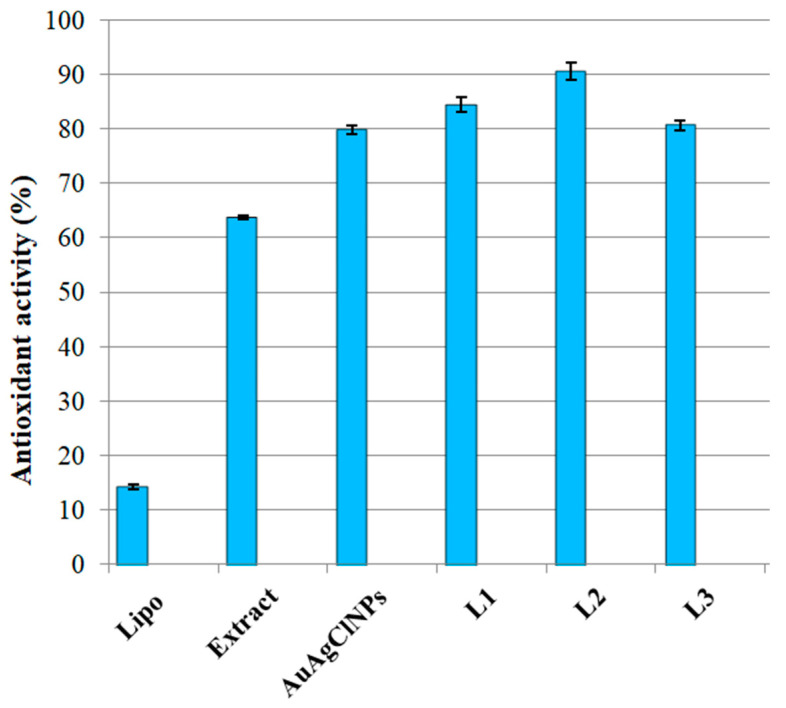
Antioxidant activity of the obtained samples estimated using the chemiluminescence technique.

The in vitro antioxidant activity assessed by the chemiluminescence (CL) technique revealed the antioxidant properties of the samples (Figure 6). The biohybrids showed the greatest values of AA% (between 80.62 and 90.48%), while the bio-inspired lipid vesicles presented the lowest antioxidant activity (14.26%). Biohybrid L2 proved to be the best antioxidant system (AA = 90.48%). It is well known that vegetal extracts show free radical scavenging properties due to the presence of many bioactive molecules like phenolics (see Section 2.1 and Section 2.2). The main phenolics found in *A. millefolium* inflorescence are caffeoylquinic acids, neochlorogenic acid, chlorogenic acid, and caffeic acid [46], which are responsible for antioxidant behavior. The phytogenically metallic particles prepared in this work presented a higher value of AA% compared to the extract precursor due to their nanosize and their composition. These results are in line with our previous studies [17,24]. The potentiation of the antioxidant activities of biohybrids is observed due to the presence of biomimetic lipid membranes. These findings agree with our previous reports [17,24].

Statistical tests regarding antioxidant activity were performed using MATLAB R 2024a to compute the ANOVA (Analysis of Variance) and Tukey’s test (see Figure S6 and Table S1 in the Supplementary Materials). After running the ANOVA test, we obtained the *p*-value 1.8128 × 10^−16^ (less than 0.05), which indicates that there is a statistically significant difference in the AA% across the groups.

The in vitro antimicrobial activity evaluated by the agar well diffusion method revealed the biocidal properties of phyto-AuAgClNPs and the three biohybrids against *Staphylococcus aureus* and *Enterococcus faecalis*.

The selection of *Staphylococcus aureus* and *Enterococcus faecalis* for this study was based on their clinical relevance and prevalence in various infections. *Staphylococcus aureus* is a common pathogen known to cause skin infections, respiratory infections, and bacteremia. It is frequently associated with hospital-acquired infections and has shown significant resistance to multiple antibiotics, making it a critical target for antibacterial testing [47].

*Enterococcus faecalis*, on the other hand, is a significant cause of infections within the gastrointestinal tract, urinary tract infections, and endocarditis. It is also notorious for its ability to acquire and transfer antibiotic resistance genes, posing a serious challenge in clinical settings [48,49].

Evaluating the antibacterial properties of our samples against these two pathogens provides a comprehensive assessment of their potential efficacy in treating a broad range of infections.

**Figure 7:**
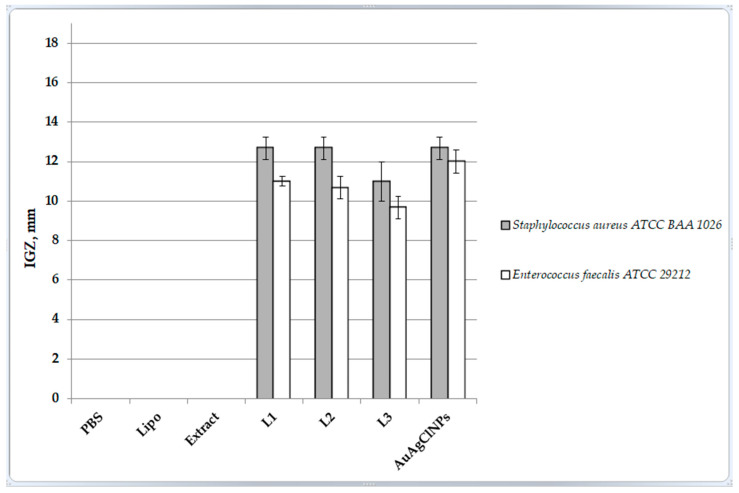
The in vitro antimicrobial activity of developed samples, expressed as diameters of the growth inhibition zone (IGZ, mm) evaluated by the agar well diffusion method.

As seen in Figure 7, only the samples containing AuAgCl NPs displayed antimicrobial efficacy against the two pathogens, but they were more effective against *Staphylococcus aureus*.

Biohybrids L1 and L2 showed similar bioactivities as yarrow-derived AuAgClNPs against the two pathogens tested. The results indicate that phyto-derived AuAgClNPs possess strong antimicrobial activity against *S. aureus* and *E. faecalis*. The incorporation of these nanoparticles into biohybrids with liposomes retains significant antimicrobial properties. Among the biohybrids, Biohybrid 2 (with a higher concentration of phyto-AuAgClNPs) demonstrated the most consistent antimicrobial effect, suggesting that the concentration of phyto-AuAgClNPs plays a crucial role in the effectiveness of the biohybrid.

Given the strong antimicrobial activity of the biohybrids, these materials hold promise for applications in medical and environmental fields where effective antimicrobial agents are essential. Further research should focus on optimizing the concentration and composition of these biohybrids to enhance their efficacy and explore their full potential.

The antimicrobial efficacy of the samples is closely related to their surface properties, as indicated by the contact angle measurements. Lower contact angles generally suggest a more hydrophilic surface, which can enhance interactions with biological environments, potentially increasing antimicrobial effectiveness.

Yarrow-derived AuAgClNPs demonstrated significant antimicrobial activity and a notable decrease in contact angle over time, indicating a hydrophilic surface that likely facilitates better interaction with bacterial cells, contributing to their antimicrobial efficacy. Despite their high initial contact angle, Chla–lecithin–liposomes (sample Lipo) become highly hydrophilic after accommodation. However, they did not show antimicrobial activity, suggesting that while surface wettability is important, the presence of antimicrobial agents is crucial. All biohybrids exhibited antimicrobial activity, with Biohybrid 2 having the strongest effect against *S. aureus* and Biohybrid 3 the lowest. The contact angle measurements support this, as Biohybrid 3 showed the highest initial contact angle, which might correlate with its relatively lower antimicrobial activity. The dynamic change in contact angle for Biohybrid 2 indicates a rapid transition to a hydrophilic state, possibly enhancing its antimicrobial properties. Aqueous vegetal extract of yarrow, having a moderately low contact angle, did not exhibit antimicrobial activity, indicating that its surface properties alone are insufficient for antimicrobial efficacy without active agents.

The results indicate that while surface wettability (as indicated by contact angle measurements) plays a significant role in enhancing antimicrobial activity, the presence of bioactive components, such as “green” AuAgClNPs, is essential. The integration of these nanoparticles into biohybrids with liposomes retains significant antimicrobial properties and highlights the importance of both chemical composition and surface properties in the development of effective antimicrobial agents. Further optimization and understanding of these properties can lead to the development of more efficient and targeted antimicrobial therapies.

To claim significant antimicrobial activity, a significant experiment has been performed for the data in Figure 7. MATLABR2024a was used to compute the ANOVA (Analysis of Variance) and Tukey’s test (see Figure S7 and Table S2 in the Supplementary Materials). ANOVA tests show if there is any significant difference in antimicrobial activity across the different experiments (groups). If the *p*-value from the ANOVA is less than 0.05, it can be concluded that there is a significant difference in the means of antimicrobial activity between at least two groups. ANOVA’s *p*-value is less than 0.05, which means there is a statistically significant difference in the antimicrobial activity across the groups. Because the ANOVA shows significant differences, Tukey’s test was used to identify which specific groups are significantly different from each other. It can be determined if specific groups (experiments) have higher antimicrobial activity compared to the others. After executing Tukey’s test, the confidence intervals and mean differences have been analyzed. Tukey’s test performed using the multcompare MATLAB procedure for the *S. aureus p*-value is 3.27 × 10^−15^ < 0.005, and for *E. faecalis*, the *p*-value is 8.24 × 10^−18^ < 0.005. The ANOVA *p*-value is less than 0.05, which means it can be proceeded with Tukey’s test to see which experiments are significantly different. If the confidence intervals in the table/matrix do not include zero, it indicates that the antimicrobial activity between the two groups is significantly different. In conclusion, the ANOVA and Tukey’s test show significant results, and we can conclude that there is significant antimicrobial activity for the experiments with non-zero values (experiments 4 through 7). From the results in Table S2, it can be confidently stated that experiments 4, 5, 6, and 7 exhibit significant antimicrobial activity compared to the others.

The IGZ values obtained for our particles are in line with those recorded by the research team of Vitalini [50], who reported inhibition zone diameters ranging from 8 to 15 mm against *Staphylococcus aureus* and 11 mm against *Enterococcus faecalis* for methanol, dichloromethane, and petroleum ether extracts of *Achillea moschata*.

**Figure 8:**
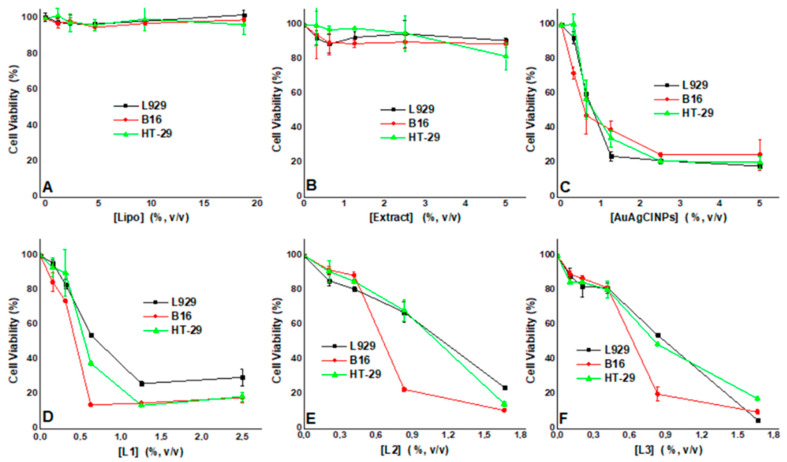
Cell viability curves recorded for all samples: (**A**)—Lipo, (**B**)—Extract, (**C**)—AuAgClNPs, (**D**)—L1, (**E**)—L2, (**F**)—L3, following 24 h of treatment for the three cell lines: L929, B16, and HT-29.

**Figure 9:**
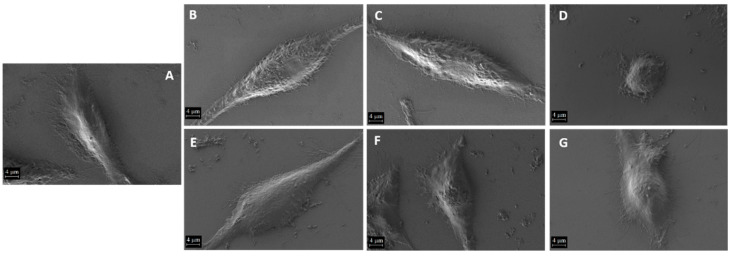
Morphological evaluation by the SEM of L929 cells grown in different conditions for 24 h: (**A**) control cells and cells treated with liposomes, (**B**) the extract, (**C**) AuAgClNPs, (**D**) L1, (**E**) L2, (**F**) and L3 (**G**). The scale bar is 4 µm.

The cell viability results for all three cell lines tested are reported in Figure 8 for 24 h and in Figure 9 for 48 h of treatment. As observed, following a 24 h treatment with the liposomes and the extract, the effects against cell viability are not important; for all concentrations tested, the cells’ viability is above 80% (Figure 8A,B).

However, when the NPs were applied, the cells’ viability decreased with increasing concentrations, as presented in Figure 8C–F. We also found different effects induced by the treatments on the three cell lines investigated.

If, for the AuAgClNPs (Figure 8C), there is almost no specificity for the cell lines, as all are similarly affected when treated with the L1–L3 samples, we found a higher specificity for the B16 cells compared with the other two cells lines (Figure 8D–F). For all samples, the toxicity effects are observed at concentrations higher than 0.5%, *v*/*v*. In order to better evaluate the effects of the treatment, for all conditions, we determined the IC_50_ values, which are reported in Table 2.

For a 48 h treatment, we still observed no toxic effect for the liposomes (Figure S8A, Supplementary Materials); however, slight toxicity was observed for the extract against the HT-29 cells at concentrations higher than 1%, *v*/*v* (Figure S8B, Supplementary Materials). For the following conditions, similar results were observed with the 24 h treatment.

Table 2 presents the IC_50_ values and the therapeutic index (TI) for both treatment times investigated (24 and 48 h). A TI higher than one is indicative of a higher specificity of the NPs for the cancer cells. For the liposomes and the extract, we could not find an IC_50_ value (ND), so we did not determine a TI.

For the AuAgClNPs at both times investigated, we found a TI smaller than 1 for HT-29, indicating no specificity for this cell line compared to the normal fibroblast, while for the B16, a slightly higher TI of 1.14 and 1.28, respectively, was determined.

However, the TI values indicate that all the hybrid samples presented an antitumoral effect against the two cancer cell lines (HT-29 and B16). The presence of a bio-matrix (biomimetic structures) in the biohybrids decreased the toxicity of yarrow-derived AuAgCl NPs against healthy cells L929 and increased the toxicity against cancer cells.

It was observed that *Alchillea*-derived samples are more effective against B16 cancer cells.

We also investigated the hemolytic effect of the NPs against the red blood cells taken from rabbits according to the ASTM F756 [51]. The results show that at concentrations higher than 1% (*v*/*v*), all the samples are hemolytic, indicating that for system use, smaller concentrations are needed. A comparison of our findings with other research results was difficult due to the fact that the same vegetal material (*Achillea millefolium* L.) may vary in terms of bioactivity due to the different chemical composition, depending on the region where the plant was collected. For L929 cells, AuNPs and AgNPs show toxicity at concentrations varying higher than 1 µg/mL depending on the formulations used [52,53,54]. HT-29 cells showed a higher resistance to NPs, and previous studies showed that higher concentrations are needed to see an increased toxicity [55,56]. Comparing our results, we see that the hybrids show a better efficiency against the colon cancer cells compared with the fibroblast. Some studies also indicate that depending on the size and types of NPs, no toxicity was observed against B16 cells at small concentrations ranging from ng/mL up to tens of µg/mL [57,58,59]. Our results show that the formulation proposed shows a higher specificity for the B16 cells compared to L929 cells.

Morphological evaluation of the cells following a 24 h treatment is presented in Figure 9. First, we evaluated L929 cell morphology for all conditions compared with the control cells (Figure 9A). As one can see, control L929 cells exhibit the specific morphology of fibroblasts with an elongated body and are well attached to the substrate due to the many filipodia that are present. The morphology is similar to other studies reporting L929 cells [60,61]. Similar results are observed for the cells treated with the lip (Figure 9B) and the extract (Figure 9C). However, when cells were treated with the NPs, various morphologies were observed. The cells treated with AuAgClNPs showed morphology changes with a dramatic reduction in the cell body, which is rounded with less filipodia (Figure 9D). Also, small traces of NPs can be found on the cells and around them. For the cells treated with the biohybrids (Figure 9E,F,G), the changes are less pronounced; however, compared with the control cells, we see fewer filipodia and also traces of NPs.

Figure S9 (Supplementary Materials) presents the B16 cells following a 24 h treatment. We can see that similar to L929 cells, the control cells and the ones treated with the liposomes and the extract show no changes in morphology (Figure S9A–C, Supplementary Materials). The morphology of the cells is similar to previously reported data, with a fibroblast-like characteristic [62,63]. For the treated cells, in addition to a significant reduction in the number of the cells still attached, we can see that the NPs start to accumulate on the surface of the cells, with some cases where the morphology is not much altered. The most dramatic change was observed for the cells treated with L3 (Figure S9G, Supplementary Materials).

For HT-29 cells, SEM images are presented in Figure S10 (Supplementary Materials). Control cells and the cells treated with liposomes and the extract show similar morphologies, with intact cell bodies and clustered cells with no changes on the membrane, as observed previously [64]. When treated with the NPs, the size of the cells decreases and for some conditions, the cells are not clustered, with NPs deposed on the membranes and around the cells. The most affected cells follow the L2 treatment.

The anticancer mechanism of the biohybrids developed in this study involves several pathways. Although AuAgClNP and biohybrid uptake by the cells used were not evaluated, there are previous studies reporting on NP uptake by different cells [65,66]. When considering the effects produced by NPs, several factors such as shape, size, zeta potential, coatings, and vehicle have to be considered [66]. It was shown that Ag-NPs can form aggregates due to their small size, entering through endocytosis into eukaryotic cells. Here, the NPs can reach the nucleus, the membrane, or other organelles, causing toxicity and inducing mitochondrial alteration, lactate dehydrogenase release, cell cycle arrest, reactive oxygen species formation, and apoptosis induction [67]. Contrary, AuNPs are incorporated easily into cells and also have anticancer properties. For this mechanism, AuNPs target cancer cells and the tumor suppressor genes and oncogenes to induce the expression of caspase-9, which is an initiator caspase involved in apoptosis [67].

Based on our findings, we suppose that the NP uptake can be related to its size. Compared to previous reports, Ag and Au NPs have smaller sizes compared to AuAgNPs and can vary from tens of nm to hundreds of nm [68,69,70]. Similar values were found for our systems as well as the NP aggregation, which can lead to NP accumulation, which previously was reported to be by caveolae-mediated endocytosis or macro-pinocytosis [71]. Based on our results, we can also state that although there is a concentration- and time-dependent effect on the cells, there is not a large difference between the biohybrids, indicating a similar mechanism.

In addition, the gold–silver chloride nanoparticles (AuAgCl NPs) green synthesized in this study using *Achillea millefolium* extract possess unique properties that contribute to their anticancer effects. Thus, the phytochemicals from the extract, such as polyphenols, act as reducing and stabilizing agents, enhancing the biocompatibility and targeting capability of the nanoparticles. *Achillea millefolium* has been reported to possess antiproliferative activity [72] due to the chemical composition rich in bioactive compounds [2,6,7]. The cytoarchitecture of HT-29 and B-16 cancer cells changed upon treatment with AuAgCl NPs and the biohybrids. Once internalized by cancer cells, the nanoparticles can disrupt the cellular membrane and interfere with mitochondrial function, further promoting cell death. Moreover, the lipid component in the biohybrids can help the uptake of biohybrids by the cancer cells.

A comparison of our findings with other research results was difficult due to the fact that the same vegetal material (*Achillea millefolium* L.) may vary in terms of bioactivity due to the different chemical composition, depending on the region where the plant was collected. Moreover, our biohybrids possess a certain composition that was not found in the literature.

The presence of lipid vesicles resulted in boosting the biohybrids’ bioperformances. This behavior agrees with our previous findings [17,24].

The biohybrids with the highest content of *Achillea*-derived AuAgClNPs (sample L2) proved to be the most stable particles with the best bioactivities. This formulation was also the most potent against the two cancer cell lines (HT-29 and B16), and it affected the healthy cells the least compared to the other hybrids.

## 3. Materials and Methods

Folin–Ciocâlteu’s phenol reagent, 3,4,5-trihydroxybenzoic acid (gallic acid, >97.5% purity), sodium carbonate (≥99.0% purity), hydrogen peroxide ≥30%, 5-Amino-2,3-dihydro-1,4-phthalazinedione (luminol, ≥97.0% purity), and buffer TRIS-HCl were provided by Sigma-Aldrich (U.S.A). Soybean lecithin (min. 97%) was purchased from Carl Roth GmbH (Karlsruhe, Germany).

Aqueous solutions were prepared using Millipore ultrapure water (electrical conductivity < 0.055 µS/cm and a resistivity of 18.22 MΩ × cm at 25 °C) obtained from a Mili-Q water system (Direct-Q 3UV system), (Molsheim, France) Merck KGaA, Darmstadt, Germany).

The vegetal material used in this research was commercial *Achillea millefolium* L., obtained from FARES: S.C. Romania, Orăștie, organic culture, harvested in 2023 from Orăștie, in the Hunedoara area, Romania, Europe (45°51′ N 23°12′ E).

The antimicrobial activity of the samples was evaluated against two pathogenic Gram-positive bacteria, including *Enterococcus faecalis* ATCC 29212 and *Staphylococcus aureus* ATCC BAA 1026. These bacterial cultures were stored at 4 °C. All chemicals used in the antibacterial investigations were sourced from VWR (Darmstadt, Germany).

### 3.1. Preparation of the Plant Extract

The aqueous herbal extract from *Achillea millefolium* L. inflorescence was obtained by the Soxhlet extraction technique [73]. Briefly, an amount of 100 g of the plant material dried and ground to the consistency of fine powder was extracted in 1000 mL solvent (ultrapure deionized water) at an extraction temperature of t > 80 °C for three hours.

The obtained extract was filtered using filter paper (Whatman no. 1, Merck KGaA, Darmstadt, Germany) and stored at 4 °C for further use.

### 3.2. Bio-Preparation of AuAgClNPs

In a beaker containing 50 mL of aqueous extract of *Achillea millefolium* L., AgNO_3_ was introduced, until its concentration reached the value of 3.7 mM. This solution was kept under continuous stirring in the dark for 24 h (VIBRAX stirrer, Milian, OH, USA, 200 rpm). It was observed that the color of the mixture turned from green-yellowish to brown, highlighting the formation of silver chloride nanoparticles. After completing this bio-reaction, an appropriate amount of HAuCl_4_ was added until the final concentration reached the value of 7.4 mM. The mixture was continuously stirred in the dark (VIBRAX stirrer, Milian, OH, USA, 200 rpm), and the solution color became purple. The phyto-molecules present in the yarrow extract helped to form the bimetallic nanoparticles by giving up electrons. The yarrow extract played a dual role as a bioreducing and capping agent for NP synthesis.

### 3.3. Preparation of Biohybrids Based on Yarrow-Derived AuAgClNPs and Artificial Cell Membranes

Artificial cell membranes were prepared from soybean lecithin according to the method described in [17]. Chlorophyll *a* (Chla) extracted from spinach leaves in our laboratory was inserted in biomimetic membranes as a spectral sensor. The biohybrids were obtained by vigorous stirring of mixtures of liposomes with AuAgClNPs in a volume ratio of 1:1, 1:2, and 2:1. These hybrids were then subjected to ultrasound irradiation in a water bath (BRANSON 1210, Marshall Scientific, Hampton, NH, USA) for 60 min (with a break after 30 min).

**Figure 10:**
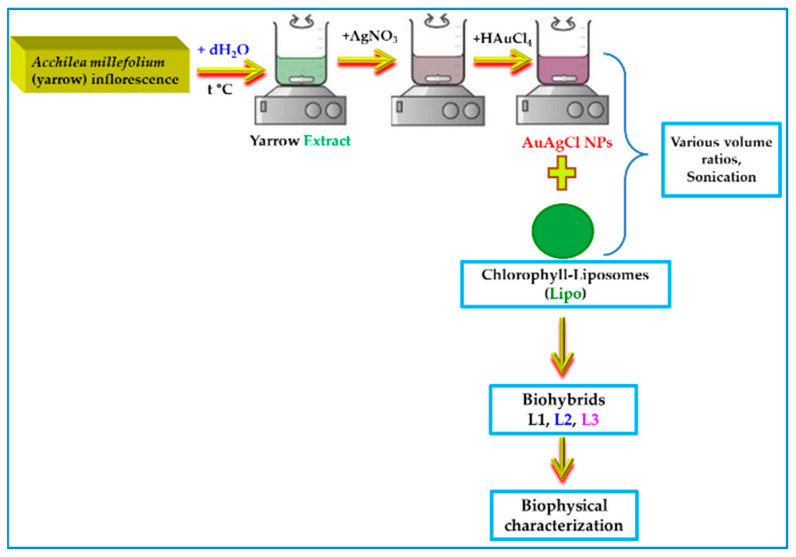
Schematic representation of the phyto-design of biohybrids containing yarrow-derived AuAgClNPs. The figure was created with Chemix (https://chemix.org/, accessed on 27 September 2024), PowerPoint (Windows 10 version), and Paint 3D (Windows 10 version), & Office 365.

The description and the abbreviation of the samples obtained in this research work are displayed in Table 3. Figure 10 suggestively depicts the process of biohybrid preparation.

### 3.4. Determination of Total Phenolic Content (TPC) of Phyto-Developed Samples

The total phenolic content (TPC) of the *Achillea millefolium* L. extract and the particles derived from it was analyzed according to the protocol described in the European Pharmacopoeia using the Folin–Ciocâlteu colorimetric method [26,74,75]. Briefly, an aliquot of the extract was mixed thoroughly with 5 mL Folin–Ciocâlteu reagent and 10 mL of a saturated solution of sodium carbonate (7.5% *w*/*v*). The mixture was stirred for 15 s and allowed to stand in the dark for 60 min, and then absorbance was measured at 760 nm using a JASCO UV-VIS V-530 spectrophotometer (Jasco International Co., Ltd., Tokyo, Japan). The total phenolic content was calculated using a gallic acid calibration curve (R^2^ = 0.9984), and the results were expressed as mg gallic acid equivalent/g dry extract (mg GAE g^−1^). Samples were analyzed in triplicate.

### 3.5. Physicochemical Characterization of Yarrow-Derived Biohybrids

The UV–Vis absorption spectra of the samples were recorded on a double beam Lambda 2S Perkin Elmer UV–Vis spectrophotometer (Perkin Elmer, Waltham, MA, USA) from 200 to 800 nm at a resolution of 1 nm.

The fluorescence emission spectra of chlorophyll-labeled samples were collected using a LS55 Perkin Elmer fluorescence spectrometer (Perkin Elmer, Waltham, MA, USA) in the wavelength range of 600–800 nm by illuminating the samples with 430 nm excitation light. The excitation and emission slit widths were set at 6 and 5 nm, respectively. The scan speed was 500 nm/min.

Fourier-transform infrared (FTIR) spectroscopy (Perkin Elmer-Spectrum 100, Waltham, MA, USA) was used in order to confirm the chemical structure of the materials. The FTIR spectra were acquired in the 4500–500 cm^−1^ range with a resolution of 4 cm^−1^ in transmittance mode.

X-ray diffraction (XRD) measurements were performed in order to investigate the crystallinity of the samples using Bragg–Brentano mode X-ray diffraction in the 2θ range from 10 to 90° with a 2θ step of 0.02° and a 2 s counting time. A Bruker D8 Advance diffractometer (Billerica, MA, USA) with CuKα (λ = 1.5418 Å) radiation (Kβ radiation was removed using a nickel filter) was used. The obtained XRD data were processed using “Bruker Diffrac plus Basic Package Evaluation v.12” and ICDD PDF4+ database 2023 for phase identification.

X-ray photoelectron spectroscopy (XPS). To determine the chemical states of the components, X-ray photoelectron spectroscopy (XPS) measurements were conducted using a SPECS photoelectron spectrometer equipped with a PHOIBOS 150 analyzer (Berlin, Germany). The X-ray source, XR-50, operates with an Al anode (hν = 1486.7 eV) at 300 W, 24 mA, and 12.5 kV. Data were acquired with a pass energy of 20 eV for individual spectra and 50 eV for the extended spectrum.

Transmission Electron Microscopy (TEM) investigations were carried out in order to determine the morpho-structural characteristics of the analyzed sample. For this, an analytical probe-corrected microscope (JEOL JEM ARM200F, (JEOL Ltd., Tokyo, Japan) operated at an acceleration voltage of 200 kV and equipped with an energy-dispersive X-ray (EDX) spectrometer (JEOL JED-2300T), (JEOL Ltd., Tokyo, Japan) was used. A droplet from each of the samples in the suspension was then deposited onto a 400-mesh carbon lacey TEM Cu grid and allowed to dry at room temperature.

Contact angle measurements. A thin liposome layer was formed by the drop-casting method. After the deposition of the drop on a silicon wafer, the sample was introduced in a vacuum oven (10^−1^ mbar) for one hour using a Varian SH100/110 (Memmingen, Germany) dry scroll pump. The liposome layers were used for contact angle measurements after the preparation. By measuring the water contact angle (CA), the hydrophobic/hydrophilic characteristics of the sample were investigated. First, films were obtained by the drop-casting method. Thus, a large drop of each sample was placed on a silicon surface. Then, the silicon wafer was vacuumed at a pressure of 10^−1^ mbar to remove the liquid matrix, thereby obtaining a film on the surface of the silicon wafer. The contact angle was further measured on the resulting film. Static CAs were measured with a drop shape analysis system, model DSA100 (Krüss GmbH, Hamburg, Germany). The samples were placed on a table under the tip of a blunt-ended stainless steel needle (0.5 mm outer diameter). The needle was attached to a syringe controlled by the DSA3^®^ PC software (V1.6-02) and used to drop the test liquid (water) onto the obtained films as well as for CA evaluation. The droplet volume was ~1 μL. For each individual sample, the analysis was performed on two different areas on the film surfaces. The value of the contact angle is obtained by fitting the experimental profile of the drop with the equation of a second-degree polynomial or with the equation of the circle. The angle between the video camera and the sample plane was ∼2°. All wetting tests were performed at room temperature.

The average size (Zav) of particles was evaluated, in triplicate, by dynamic light scattering (DLS), and the results were reported as the mean values ± S.D. DLS measurements were carried out on a Zetasizer Nano ZS (Malvern Instruments Inc., Worcestershire, UK) at a room temperature of 25 °C using 173° backscatter angle detection, assuming a laser beam at a wavelength of 633 nm as the indicator. The size distribution’s width was also determined by the means of the polydispersity index, PdI.

Zeta potential (ξ, mV) measurements were performed in triplicate at a room temperature of 25 °C in an appropriate device of the Zetasizer Nano ZS (Malvern Instruments Ltd., Malvern, UK) by applying an electric field across the analyzed samples. The ξ measurements were carried out in ultrapure water (pH 6.98), and the ξ values were reported as mean ± S.D.

### 3.6. Bio-Investigation of Yarrow-Developed Hybrids

#### 3.6.1. In Vitro Antioxidant Activity Evaluation

The chemiluminescence (CL) method was applied using luminol H_2_O_2_ as a generator system in a TRIS-HCl buffer at pH 8.75 using a Sirius Luminometer ( Berthold Technologies GmbH & Co. KG, Bad Wildbad, Germany). The antioxidant activity AA% of the samples was calculated using the following relation:

(1) $$\mathrm{AA}\;(\%)=\frac{I_{0}-I_{S}}{I_{0}}\times 100$$

where I_0_ = the maximum CL intensity for reference at t = 5 s and Is = the maximum CL for sample at t = 5 s [76]. All measurements were replicated three times.

#### 3.6.2. In Vitro Antibacterial Activity Analysis

Stock cultures of bacteria (*Staphylococcus aureus* and *Enterococcus faecalis*) were sub-cultured onto Luria Bertani Agar acc. Miller (LBA) plates and incubated at 37 °C. To assess the antibacterial properties of the tested samples, the agar well diffusion method, as previously described [24], was employed. In brief, the surface of the LBA was inoculated by spreading a specific volume of the bacterial inoculum (1 mL). Wells were then created using a sterile 6 mm diameter Durham tube and filled with 50 μL of each sample. The plates were incubated at 37 °C for 18–24 h. The antimicrobial agent diffused into the LBA, preventing the growth of the test bacteria, and the diameters of the resulting IGZ were subsequently measured according to Ponce et al. [77]. The standard deviation (SD) was computed by taking the square root of the variance using the STDEV function in Excel 2010.

#### 3.6.3. In Vitro Cytotoxicity Assay

Cell Culture Conditions

L929 fibroblast cells, B16 mouse melanoma cells, and HT-29 human adenocarcinoma cells (ATCC, Manassas, VA, USA) were grown in DMEM (Dulbecco’s Modified Eagle Medium) supplemented with 2 mM L-Glutamine, 10% fetal calf serum (FCS), 100 units/mL of penicillin, and 100 µg/mL of streptomycin at 37 °C in a humidified incubator under an atmosphere containing 5% CO_2_. All cell cultivation media and reagents were purchased from Biochrom AG (Berlin, Germany) and Sigma-Aldrich (Darmstadt, Germany).

Cell Viability Assay

Cell viability following 24 and 48 h post-treatment was evaluated using 3-(4,5-dimethylthiazol-2-yl)-2,5-diphenyltetrazolium bromide (MTT) assay. The cells were seeded in 96-well plates (7000 cells/well for L929 and B16 cells and 14,000 cells/well for HT-29) and cultured for 24 h in the medium. The following day, the medium was changed, and the investigated samples were added in incubated for the desired time. The negative control was represented by cells cultivated in the medium without the investigated compounds. Following the desired incubation time, the medium was changed to 1 mg/mL MTT solution and incubated for an additional 4 h at 37 °C. The medium was finally collected, and DMSO was added to dissolve the insoluble formazan product. The absorbance of the samples was recorded at 570 nm using a plate reader, Mithras 940 (Berthold). The data were corrected for the background, and the percentage of viable cells was obtained using the following equation:

Cell viability = [(A^570^ of treated cells)/(A^570^ of untreated cells)] 100% (2)

When possible, the half-maximal inhibitory concentration (IC50) was obtained by fitting the experimental data with a logistical sigmoidal equation using Origin 8.1 software from Microcal Inc. (Los Angeles, CA, USA). The therapeutic index (TI), which is seen as a quantitative value reporting on the affinity of the treatment for the cancer cells, was determined according to the following equation:

TI = ED50/TD50 (3)

where ED50 represents the IC50 values of the treatment against cancer cells (treatment efficiency) and TD50 represents the IC50 values of the treatment against normal cells (treatment toxicity).

Cell fixation for SEM

For SEM recordings, the cells were seeded on glass slides and treated with the compounds investigated for 24 h at a concentration close to IC50 values. Following the cell treatment, the slides were washed with PBS and first fixed for 10 min with 3% formaldehyde and 0.2% glutaraldehyde, followed by a 20 min post-fixation, using 0.1% OsO_4_. The morphology of the samples was investigated using a Gemini 500 Carl Zeiss Field Emission Scanning Electron Microscope (FESEM) working in both High Vacuum (HV) and Variable Pressure (VP) modes, from 0.2 to 30 kV, equipped with LaB6 filament, NanoVP mode, InLens, and SE2 detectors.

## 4. Conclusions

This study described an original biostrategy to prepare multifunctional biohybrids based on biomimetic membranes and yarrow-derived AuAgClNPs.

This research emphasizes the importance of valorization of phyto-waste for the development of “green” bioactive bio-entities.

Optical studies, including UV-Vis, emission fluorescence, and FTIR, confirmed the formation of biohybrids. The involvement of phytocompounds arising from *A. millefolium* (such as carboxylic acids, alcohols, polyphenols, ethers, etc.) was also highlighted.

Moreover, the XRD results were well correlated with the FTIR, EDX, and XPS spectra regarding the particles’ phase.

The particles obtained in this study showed moderate and good stability. DLS measurements and TEM confirmed the nanoscale size of the phytogenically derived particles.

The biological activities of biohybrids were closely related to their wetting properties, their AuAgCl NP content, and the presence of active biomolecules on the surface of these metallic particles. Contact angle measurements revealed the hydrophilic properties of the samples. By adding in a biological matrix (artificial cell structures), the phyto-derived AuAgCl NPs presented improved bioactivities and biocompatibility.

Cells treated with these phyto-prepared hybrid nanoparticles displayed various morphological changes and behaviors.

The best results, including good physical stability, antioxidant properties, antimicrobial action, and antiproliferative activity, were observed in the biohybrid with the highest AuAgClNP content (L2).

The presence of bio-inspired lipid layers in the composition of biohybrids played a key role in boosting the bioactivities and their stability.

Future studies will be performed to improve the composition of these biohybrids in order to achieve improved bioactivities.

Bio-inspired strategies for developing nanoparticles are currently of significant interest. By integrating inorganic components into biomimetic platforms, biohybrids with enhanced bioactivities and reduced cytotoxicity can be produced.

As humans, we have the obligation to protect nature. We must act according to the 12 principles of Green Chemistry and use natural raw materials in our technologies to minimize waste and avoid pollution. In this way, we will keep nature always green, and we humans will always be able to benefit from the therapeutic treasure of the Plant Kingdom.

## Figures and Tables

**Table 1:** Determination of total phenol content (TPC).

Sample Abbreviation	Determination of Total Phenol Content (TPC) mg GAE·g^−1^
Extract	39.84 ± 0.65
AuAgClNPs	4.02 ± 0.11
Lipo	-
L1	1.63 ± 0.29
L2	2.89 ± 0.76
L3	0.94 ± 0.18

Abbreviations: extract = aqueous vegetal extract of yarrow (*Achillea millefolium* L.); AuAgClNPs = gold–silver chloride NPs phytosynthesized using yarrow extract; Lipo = chlorophyll–lecithin–liposomes; L1 = Biohybrid 1 (Lipo: Phyto-AuAgClNPs = 1:1, *v*/*v*); L2 = Biohybrid 2 (Lipo: Phyto-AuAgClNPs = 1:2, *v*/*v*); L3 = Biohybrid 3 (Lipo: Phyto-AuAgClNPs = 2:1, v/v); GAE = gallic acid.

**Table 2:** Half inhibitory concentrations (IC_50_) and the therapeutic index (TI) for the experimental conditions investigated at 24 and 48 h of treatment.

Sample	24 h					48 h				
	IC_50_/%, *v*/*v*			TI		IC_50_/%, *v*/*v*			TI	
	L929	HT-29	B16	HT-29	B16	L929	HT-29	B16	HT-29	B16
Lipo	ND	ND	ND	-	-	ND	ND	ND	-	-
E	ND	ND	ND	-	-	ND	ND	ND	-	-
AuAgClNPs	0.79	0.88	0.69	0.89	1.14	0.68	0.75	0.53	0.91	1.28
L1	0.79	0.68	0.43	1.16	1.83	0.65	0.54	0.38	1.20	1.71
L2	1.16	1.05	0.66	1.10	1.75	1.09	0.93	0.49	1.17	2.22
L3	0.91	1.06	0.61	0.85	1.49	0.88	0.82	0.48	1.07	1.83

**Table 3:** The abbreviation and description of the obtained samples.

Sample Abbreviation	Description	AuAgClNP Content (%, *v*/*v*)
Extract	Aqueous vegetal extract of yarrow (*Achillea millefolium* L.)	0
AuAgClNPs	Phyto-AuAgClNPs	100
Lipo	Chla–Lecithin–Liposomes	0
L1	Biohybrid 1 (Lipo: Phyto-AuAgClNPs = 1:1, *v*/*v*)	50.0
L2	Biohybrid 2 (Lipo: Phyto-AuAgClNPs = 1:2, *v*/*v*)	66.7
L3	Biohybrid 3 (Lipo: Phyto-AuAgClNPs = 2:1, *v*/*v*)	33.3

## Data Availability

The data were included in the text.

## Supplementary Materials

The supporting information can be downloaded at https://www.mdpi.com/article/10.3390/ijms252211929/s1.
