# Supplementary material for: Bioactive Hybrids Containing Artificial Cell Membranes and Phyto-Gold–Silver Chloride Bio-Nanoparticles
Source: Int J Mol Sci. 2024 Nov 6;25(22):11929. doi: 10.3390/ijms252211929 (PMC11593899; doi:10.3390/ijms252211929)
Supplement: Supplementary file 1 [file ijms-25-11929-s001.zip › ijms-3263026-supplementary.pdf]

Supplementary material

Bioactive Hybrids Containing Artificial Cell Membranes and Phyto-Gold-Silver Chloride Bio-Nanoparticles

Marcela-Elisabeta Barbinta-Patrascu [1], Cornelia Nichita [2,3,*], Monica Enculescu [4], Valentin-Adrian Maraloiu [4], Mihaela Bacalum [5], Camelia Ungureanu [6], Catalin Constantin Negrila [4] and Irina Zgura [4,*]

[1] Department of Electricity, Solid-State Physics and Biophysics, Faculty of Physics, University of Bucharest, 405 Atomistilor Street, P.O. Box MG-11, 077125 Magurele, Romania; marcela.barbinta@unibuc.ro
[2] CTT-3Nano-SAE Research Center, Faculty of Physics, University of Bucharest, ICUB, 405 Atomistilor Street, P.O. Box MG-38, 077125 Magurele, Romania
[3] National Institute for Chemical-Pharmaceutical Research and Development, 112 Vitan Avenue, 031299 Bucharest, Romania
[4] National Institute of Materials Physics, Atomistilor 405A, 077125 Magurele, Romania; mdatcu@infim.ro (M.E.); maraloiu@infim.ro (V.-A.M.); catalin.negrila@infim.ro (C.C.N.)
[5] Department of Life and Environmental Physics, Horia Hulubei National Institute for Physics and Nuclear Engineering, Reactorului, 30, 077125 Magurele, Romania; bmihaela@nipne.ro
[6] Department of General Chemistry, Faculty of Chemical Engineering and Biotechnologies, The National University of Science and Technology POLITEHNICA Bucharest, Gheorghe Polizu 1-7 Street, 011061 Bucharest, Romania; ungureanucamelia@gmail.com
* Correspondence: cornelia.nichita@unibuc.ro (C.N.); irina.zgura@infim.ro (I.Z.)

1. *Spectral Characterization of Achillea-derived AuAgCl NPs, by UV-Vis absorption*

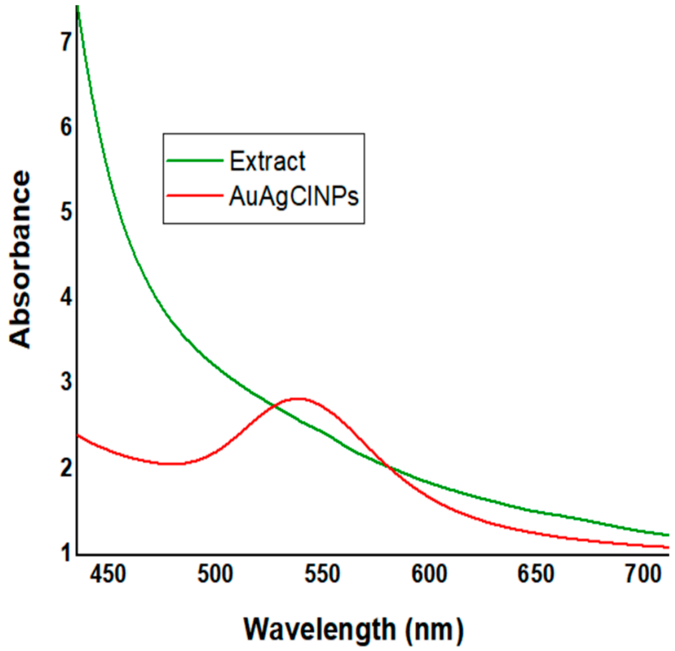

Figure S1. UV-Vis absorption spectra of yarrow-derived AuAgClNPs as compared to the extract precursor.

2. *Evaluation of Zeta Potential of the Phytometallic Particles*

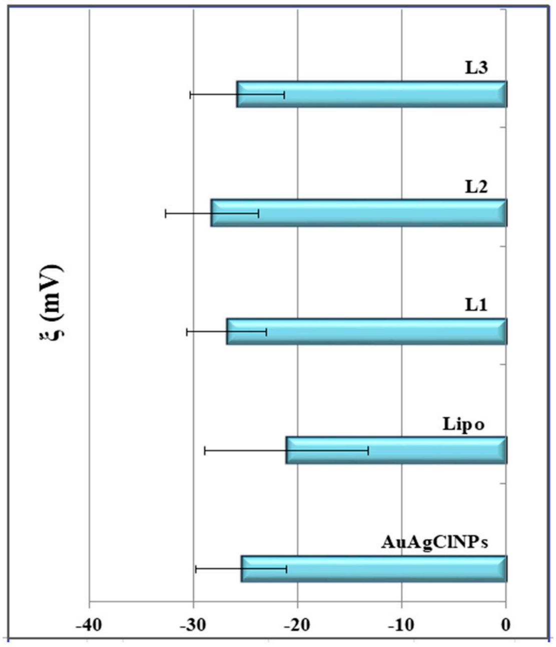

Figure S2. Comparative presentation of the electrokinetic potential of the particles developed in this study.

3. *DLS measurements*

The average particle size was estimated by DLS measurements. These values are presented in the Figure S3.

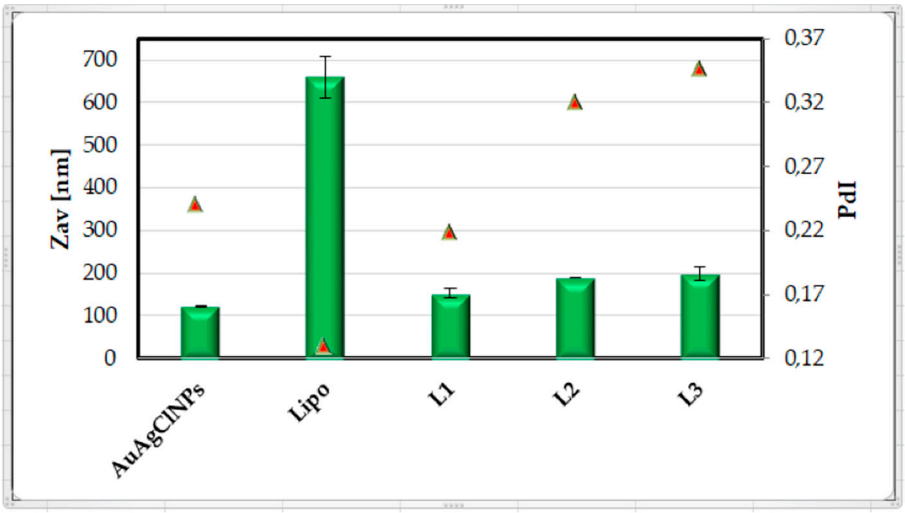

Figure S3. Comparative presentation of the average particle size (Zav, nm) and PdI index of biogenic particles, estimated by dynamic light scattering (DLS) measurements. The samples (excepting lipid vesicles) were previously ultrasonated prior DLS measurements.

The size distribution for the non-ultrasonicated liposome sample is displayed in Figure S4.

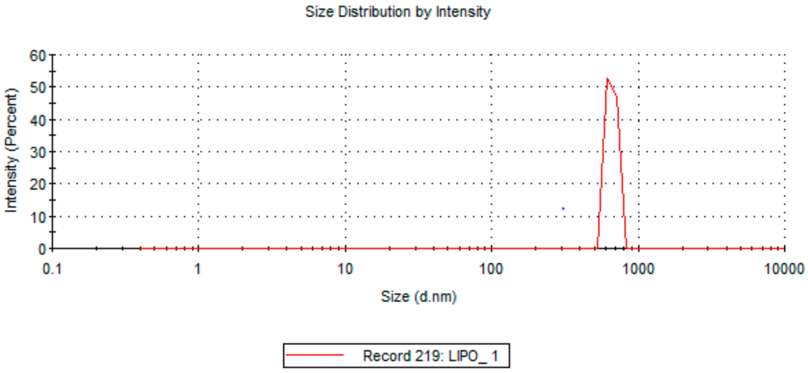

Figure S4. Size distribution profile for Lipo_1 (sample non-ultrasonicated); Zav=661±48.54 nm (100%) and PdI = 0.049.

4. Morphological and Compositional Characterization of Pristine Components of Bio-hybrids

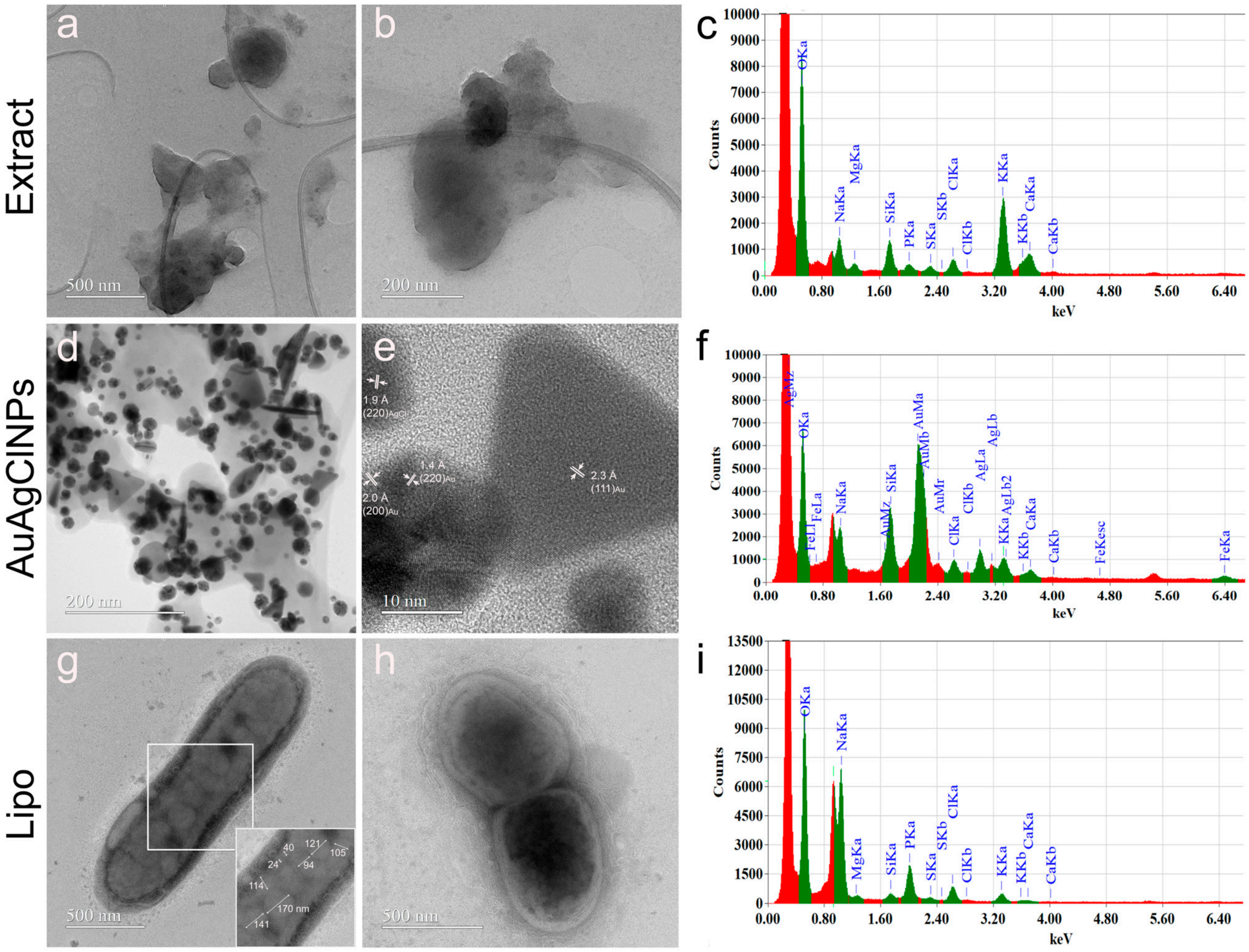

Figure S5. CTEM images (a, d, g), HRTEM images (b, e, h) and EDX spectra (c, f, i) obtained on the pristine components of biohybrids.

5. The Wetting Properties of Achillea-derived Samples

The apparent contact angle θ* in Cassie impregnation state is given by the expression:

$$\cos\theta^{*} = (1 - f_S) + f_S\cos\theta \qquad (1)$$

The apparent contact angle in the Cassie–Baxter regime can be obtained by using expression:

$$\cos\theta^{*} = -(1 - f_S) + f_S\cos\theta \qquad (2)$$

where, f_s is the fraction of the surface that is in contact with liquid under the drop in both equations. From equation (1) it can be obtained that the spreading of the liquid beyond the drop leads to a smoothing of the substrate's roughness, thus improving its wetting properties, θ* < θ [42]. Also from equation (2) it can be obtained that the surface texture could increase substrate hydrophobicity through the air trapping in the pockets of the surface.

6. Significant Antioxidant Activity

In order to claim a significant AA%, a significance test has been performed for the data in **Figure 6** in the main text in the manuscript**.** MATLAB R 2024a was used to compute the ANOVA (Analysis of Variance) and Tukey's test. ANOVA shows if there is any significant difference in AA% across the different experiments (groups). If the p-value from the ANOVA is less than 0.05, it can be concluded that there is a significant difference in the means of AA% between at least two groups.

Because the ANOVA shows significant differences, Tukey's test was used to identify which specific groups are significantly different from each other. It can be determined if specific groups (experiments) have higher AA% compared to the others.

In **Figure S6** the generic MATLAB code is presented for analyzing the AA% experiments. The results are synthetized in **Table S1**.

```
% Data: experiments and corresponding AA values
experiments_AA = [1 1 1 2 2 2 3 3 3 4 4 4 5 5 5 6 6 6]; % Experiment numbers

values_AA=[13.64 14.34 14.81 63.28 63.8 64.15 78.68 79.95 80.8 82.66 84.54 ...
    85.85 88.49 90.73 92.22 79.41 80.77 81.68]; % AA values
values = values_S_aureus;

% Perform one-way ANOVA
[p, tbl, stats] = anova1(values_AA, experiments_AA);

% Display ANOVA p-value
disp(['ANOVA p-value: ', num2str(p)]);

% If the ANOVA is significant (p < 0.05), perform Tukey's post-hoc test
if p < 0.05
    % Perform Tukey's HSD test and get the output as a matrix
    [comparison_matrix, means, h, group_names] = multcompare(stats);

    % Display Tukey's test results matrix
    disp('Tukey post-hoc test results (Confidence Intervals):');
    disp(comparison_matrix);
else
    disp('No significant differences found by ANOVA.');
end
```

Figure S6. MATLAB code for performing a significance test for the AA%.

After running the ANOVA test we obtained the p-value 1.8128e-16 (less than 0.05) that indicates that there is a statistically significant difference in the AA% across the groups.

After executing the Tukey's test, the confidence intervals and mean differences have been analyzed (**Table S1**).

Table S1. Tukey's test performed using multcompare MATLAB procedure for the AA% (p value is 1.8128e-16)

AA%, ANOVA1 p value 1.8128e-16<0.05					
**Group 1**	**Group 2**	**Mean Difference**	**Lower Bound**	**Upper Bound**	**Significance**
1	2	-52.8534	-49.48	-46.1066	0
1	3	-68.9201	-65.5467	-62.1732	0
1	4	-73.4601	-70.0867	-66.7132	0
1	5	-79.5901	-76.2167	-72.8432	0
1	6	-69.7301	-66.3567	-62.9832	0
2	3	-19.4401	-16.0667	-12.6932	0
2	4	-23.9801	-20.6067	-17.2332	0
2	5	-30.1101	-26.7367	-23.3632	0
2	6	-20.2501	-16.8767	-13.5032	0
3	4	-7.9134	-4.54	-1.1666	0.0071
3	5	-14.0434	-10.67	-7.2966	0
3	6	-4.1834	-0.81	2.5634	0.9608
4	5	-9.5034	-6.13	-2.7566	0.0006
4	6	0.3566	3.73	7.1034	0.0275
5	6	6.4866	9.86	13.2334	0

The ANOVA and Tukey's test show significant results, and we can conclude that there is significant AA% for the experiments with non-zero values (experiments 1 through 6). From the results in **Table S1** it can be confidently stated that experiments 1 through 6 exhibit significant AA% compared to the others.

7. *Significant Antimicrobial Activity*

To claim significant antimicrobial activity, a significance test has been performed for the data from **Figure 7** in the main text in the manuscript. MATLABR2024a was used to compute the ANOVA (Analysis of Variance) and Tukey's test.

Tukey's test performed using multcompare MATLAB procedure for the *S. aureus* p value is 3.27e-15<0.005) and for the *E. faecalis* p value is 8.24e-18<0.005).

In **Figure S7** the generic MATLAB code is presented for analyzing alternatively both *E. faecalis* and *S. aureus*. The results of are synthetized in **Table S2**.

```
% Data: experiments and corresponding antimicrobial activity values
experiments = [1 1 1 2 2 2 3 3 3 4 4 4 5 5 5 6 6 6 7 7 7]; % Experiment numbers

values_E_faecalis = [0.00 0.00 0.00 0.00 0.00 0.00 0.00 0.00 0.00 11.00 11.00 11.00 ...
          11.00 11.00 10.00 10.00 9.00 10.00 12.00 12.00 12.00]; % Antimicrobial activity values
values_S_aureus = [0.00 0.00 0.00 0.00 0.00 0.00 0.00 0.00 0.00 12.00 13.00 13.00 ...
          13.00 12.00 13.00 12.00 10.00 11.00 13.00 13.00 12.00]; % Antimicrobial activity values
%values = values_S_aureus;
values = values_E_faecalis;

% Perform one-way ANOVA
[p, tbl, stats] = anova1(values, experiments);

% Display ANOVA p-value
disp(['ANOVA p-value: ', num2str(p)]);

% If the ANOVA is significant (p < 0.05), perform Tukey's post-hoc test
if p < 0.05
    % Perform Tukey's HSD test and get the output as a matrix
    [comparison_matrix, means, h, group_names] = multcompare(stats);

    % Display Tukey's test results matrix
    disp('Tukey post-hoc test results (Confidence Intervals):');
    disp(comparison_matrix);
else
    disp('No significant differences found by ANOVA.');
end
```

Figure S7. MATLAB code for performing a significance test for the antimicrobial activity for *E. faecalis* and *S. aureus*.

Table S2. Tukey's test performed using multcompare MATLAB procedure for the *S. aureus* (left, ANOVA1 p value is 3.26872990001940e-15<0.005) and *E. faecalis* (right, ANOVA1 p value 8.23788570598114e-18<0.005).

S. aureus

Group 1	Group 2	Mean Difference	Lower Bound	Upper Bound	Significance
1	2	-1.4902	0	1.4902	1
1	3	-1.4902	0	1.4902	1
1	4	-14.1569	-12.667	-11.18	0
1	5	-14.1569	-12.667	-11.18	0
1	6	-12.4902	-11	-9.51	0
1	7	-14.1569	-12.667	-11.18	0
2	3	-1.4902	0	1.4902	1
2	4	-14.1569	-12.667	-11.18	0
2	5	-14.1569	-12.667	-11.18	0
2	6	-12.4902	-11	-9.51	0
2	7	-14.1569	-12.667	-11.18	0
3	4	-14.1569	-12.667	-11.18	0
3	5	-14.1569	-12.667	-11.18	0
3	6	-12.4902	-11	-9.51	0
3	7	-14.1569	-12.667	-11.18	0
4	5	-1.4902	0	1.4902	1
4	6	0.1764	1.6667	3.1569	0.0241
4	7	-1.4902	0	1.4902	1
5	6	0.1764	1.6667	3.1569	0.0241
5	7	-1.4902	0	1.4902	1
6	7	-3.1569	-1.6667	-0.176	0.0241

E. faecalis

Group 1	Group 2	Mean Difference	Lower Bound	Upper Bound	Significance
1	2	-0.8604	0	0.8604	1
1	3	-0.8604	0	0.8604	1
1	4	-11.8604	-11	-10.14	0
1	5	-11.5271	-10.667	-9.806	0
1	6	-10.5271	-9.6667	-8.806	0
1	7	-12.8604	-12	-11.14	0
2	3	-0.8604	0	0.8604	1
2	4	-11.8604	-11	-10.14	0
2	5	-11.5271	-10.667	-9.806	0
2	6	-10.5271	-9.6667	-8.806	0
2	7	-12.8604	-12	-11.14	0
3	4	-11.8604	-11	-10.14	0
3	5	-11.5271	-10.667	-9.806	0
3	6	-10.5271	-9.6667	-8.806	0
3	7	-12.8604	-12	-11.14	0
4	5	-0.5271	0.3333	1.1937	0.8308
4	6	0.4729	1.3333	2.1937	0.0017
4	7	-1.8604	-1	-0.14	0.0184
5	6	0.1396	1	1.8604	0.0184
5	7	-2.1937	-1.3333	-0.473	0.0017
6	7	-3.1937	-2.3333	-1.473	0

8. Evaluation of Biological Activities of Phyto-Particles

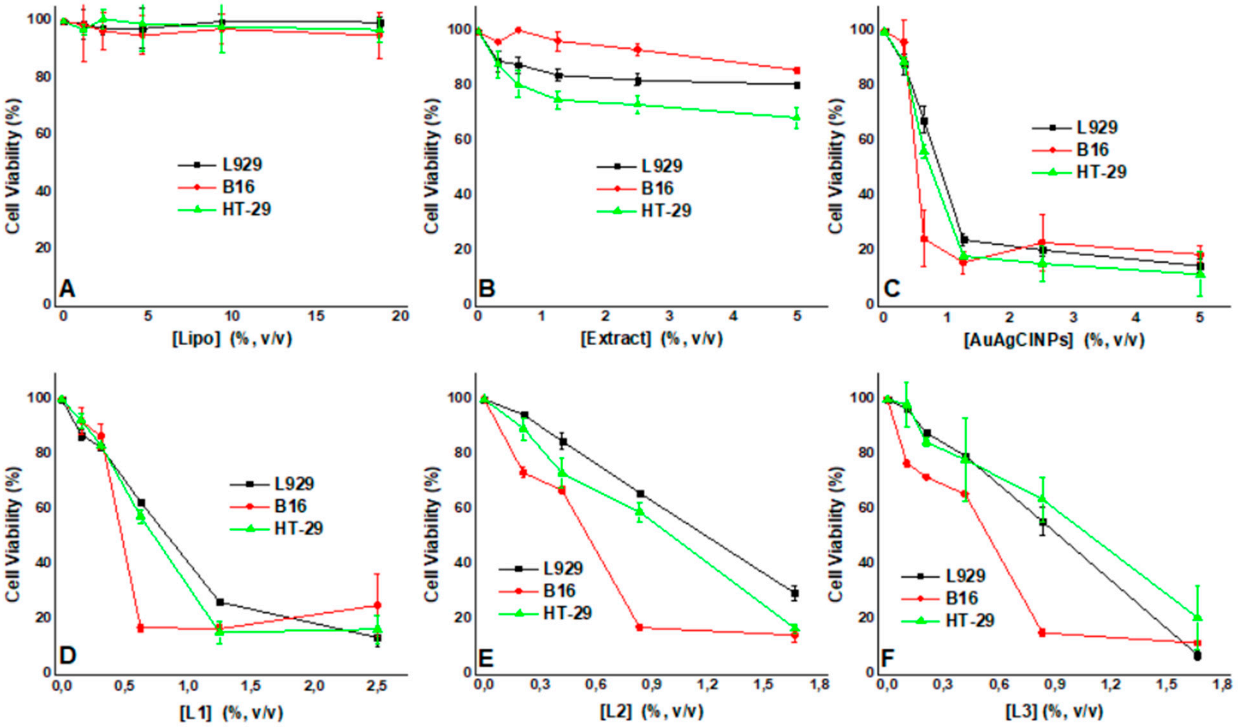

Figure S8. Cell viability curves recorded for all samples following 48h of treatment for the three cell lines: L929, B16 and HT-29.

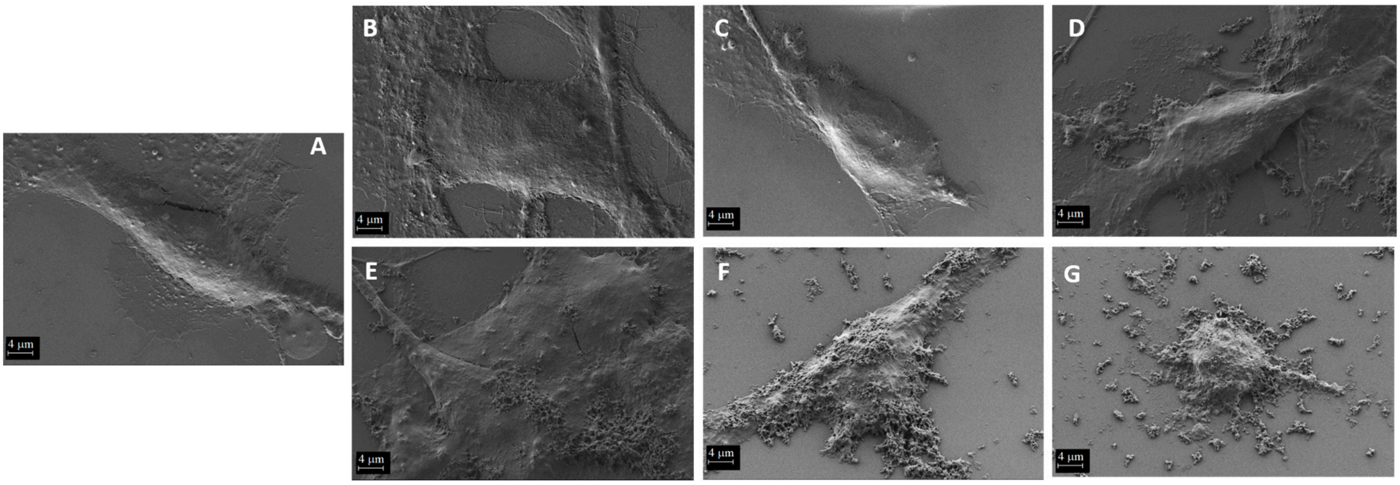

Figure S9. Morphological evaluation by SEM of B16 cells grown in different condition for 24h: A – control cells, and cells treated with liposomes (B), the extract (C), AuAgClNPs (D), L1 (E), L2 (F) and L3 (G). The scale bar is 4 µm.

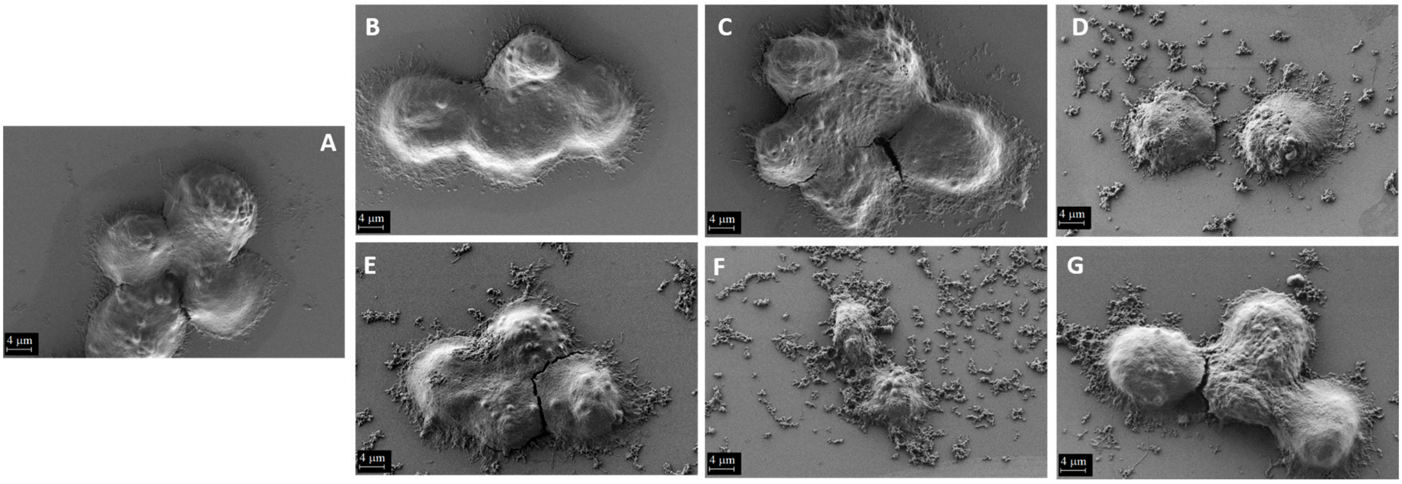

Figure S10. Morphological evaluation by SEM of HT-29 cells grown in different condition for 24h: A – control cells, and cells treated with liposomes (B), the extract (C), AuAgClNPs (D), L1 (E), L2 (F) and L3 (G). The scale bar is 4 μm.
